# Evaluation of Professional Setbacks and Resilience in Biomedical Scientists During the COVID-19 Pandemic

**DOI:** 10.1001/jamanetworkopen.2023.28027

**Published:** 2023-08-09

**Authors:** Nicole C. Woitowich, Anthony C. Waddimba, Chen Yeh, Lutfiyya N. Muhammad, Ann Marie Warren, Christine V. Wood

**Affiliations:** 1 Department of Medical Social Sciences, Northwestern University Feinberg School of Medicine, Chicago, Illinois; 2 Division of Surgical Research, Department of Surgery, Baylor University Medical Center, Dallas, Texas; 3 Division of Biostatistics, Department of Preventive Medicine, Feinberg School of Medicine, Northwestern University, Chicago, Illinois; 4 Division of Trauma, Acute Care, and Critical Care Surgery, Baylor University Medical Center, Dallas, Texas

## Abstract

This survey study evaluates whether resilience was associated with professional and career setbacks among biomedical scientists during the COVID-19 pandemic.

## Introduction

Biomedical scientists faced unprecedented challenges during the COVID-19 pandemic, analogous in many ways to their clinician colleagues.^1^ Nevertheless, insufficient attention has been paid to adverse psychological consequences of the pandemic for scientists. We investigated whether resilience was associated with scientists’ professional and career setbacks during the pandemic.

## Methods

In this survey study, we sampled recipients of National Institutes of Health funding and members of the National Postdoctoral Association. Data were collected from October 1 to November 30, 2020. Participants self-reported demographic information including gender, race, and ethnicity. Respondents answered yes or no or unsure to the question, “Have you experienced career or professional setbacks as a result of the COVID-19 pandemic?” They also completed the 10-item Connor-Davidson Resilience Scale (CD-RISC-10).^2^ This study followed the AAPOR reporting guideline and disclosure standards.^3^ The Northwestern University institutional review board approved this study, and participants provided electronic informed consent. The dependent variable was whether respondents experienced a professional setback during the pandemic. Logistic regression models of the outcome were fitted on CD-RISC-10 score, gender, additional child and/or elder care work, and professional career stage. Analyses were conducted using GraphPad Prism, version 9. Two-sided *P* < .05 was considered significant. The eMethods in Supplement 1 provides additional details.

## Results

Of 31 647 invitations, 635 individuals met the inclusion criteria and completed the survey (response rate, 2%) (Table). A total 390 respondents (61%) experienced a career or professional setback during the pandemic. The median CD-RISC-10 score was 29 (IQR, 29-32).

**Table.** Respondent Demographics

Characteristic	Respondents, No. (%) (N = 635)
Gender identity	
Female	369 (58)
Male	257 (40)
Prefer to self-describe or not say	9 (1)
Age, y	
18-24	1 (<1)
25-34	109 (17)
35-44	231 (36)
45-54	145 (23)
55-64	91 (14)
65-74	49 (8)
≥75 or prefer not to say	9 (1)
Race	
American Indian or Alaska Native	1 (<1)
Asian or Pacific Islander	58 (9)
Black or African American	10 (2)
White	528 (83)
Multiracial	17 (3)
Prefer to self-describe or not say	21 (3)
Ethnicity	
Hispanic or Latino/a/x	51 (8)
Not Hispanic or Latino/a/x	584 (92)
Highest degree obtained	
PhD	503 (79)
MD	62 (10)
MD and PhD	62 (10)
Other	8 (1)
Experienced a professional setback	
Yes	390 (61)
No or unsure	245 (39)
Took on additional care work	
Yes	305 (48)
No or unsure	330 (52)
Career stage	
Early	102 (16)
Mid to late	533 (84)

In an unadjusted model, CD-RISC-10 score was positively associated with experiencing a setback (odds ratio [OR], 1.04; 95% CI, 1.01-1.07; *P* = .008). In a multivariable model, CD-RISC-10 score (OR, 1.04; 95% CI, 1.01-1.07; *P* = .02), gender (OR, 2.50; 95% CI, 1.80-3.51; *P* < .001), and additional family care responsibilities (OR, 1.84; 1.32-2.59; *P* < .001) were associated with experiencing a setback but career stage was not (OR, 1.04; 95% CI, 0.74-1.47; *P* = .80) (Figure).

**Figure.**
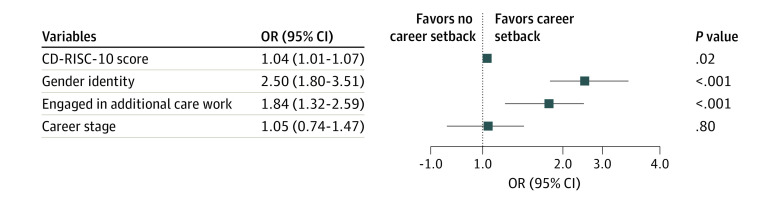
Odds of Experiencing a Professional Setback From the Logistic Regression Analysis The reference variable for gender was man. The reference variable for additional care work was No. The reference variable for career stage was mid to late. Squares indicate odds ratios (ORs), with horizontal lines indicating 95% CIs.

## Discussion

Biomedical scientists engaged in synergistic efforts to address the COVID-19 pandemic alongside health care professionals. Scientists were expected to persevere in achieving professional goals, expectations, and milestones despite facing disparate professional and personal challenges.^4^ Our data highlight the differential consequences of the pandemic for women scientists of all career stages, who experienced greater household responsibilities while facing social pressure to advance their careers.

Resilience may play an important role in how scientists experienced the pandemic. This aligns with the concept of resilience as a protective psychological factor in response to stressful experiences.^5^ These data surprisingly suggest that resilience was associated with experiencing setbacks during the pandemic. Although individual resilience is a complex and nonstatic phenomenon, it is unlikely to be a causal factor. Sociocultural factors such as a gender and/or additional family care responsibilities were also associated with professional setbacks among scientists during the pandemic.

This is one of the first studies to evaluate scientists’ resilience using the CD-RISC-10. Overall, scientists’ scores ranked below the national prepandemic average but were comparable to other postpandemic reports.^6^

Study limitations include a small-sized sample that was predominately White. Experiences of racial and ethnic minority scientists may be underrepresented. Additionally, acquiescence response or social desirability bias cannot be ruled out. Data were collected within 60 days, providing only a brief snapshot of scientists’ pandemic era experiences. Most importantly, causality could not be determined due to the cross-sectional study design.

The promotion of resiliency building may support beneficial coping skills but might not sufficiently counteract sociocultural and contextual pressures arising from gender disparities within the biomedical workforce. Focused strategies to support the professional advancement of women and gender-minority scientists, particularly in the wake of the pandemic, are warranted.

## Notes

Supplement 1.
eMethods.

Supplement 2.
Data Sharing Statement
