# Supplementary material for: Evaluation of Professional Setbacks and Resilience in Biomedical Scientists During the COVID-19 Pandemic
Source: JAMA Netw Open. 2023 Aug 9;6(8):e2328027. doi: 10.1001/jamanetworkopen.2023.28027 (PMC10413169; doi:10.1001/jamanetworkopen.2023.28027)
Supplement: Supplement 1. — eMethods. [file jamanetwopen-e2328027-s001.pdf]

Supplemental Online Content

Woitowich NC, Waddimba AC, Yeh C, Muhammad LN, Warren AM, Wood CV. Evaluation of professional setbacks and resilience in biomedical scientists during the COVID-19 pandemic. *JAMA Netw Open.* 2023;6(8):e2328027. doi:10.1001/jamanetworkopen.2023.28027

eMethods.

This supplemental material has been provided by the authors to give readers additional information about their work.

© 2023 Woitowich NC et al. *JAMA Network Open.*

eMethods

All work was done under the approval of the Northwestern University Institutional Review Board (STU00213241) and all respondents provided electronic informed consent to participate in this study.

Survey Instrument & Inclusion Criteria

An online survey instrument (Qualtrics, Provo, UT, USA) was developed to assess biomedical scientists' attitudes and opinions towards COVID-19 related research interruptions and its impact on their productivity and professional development. The instrument contained 39 items and included themes such as demographics (gender, race, ethnicity, sexual orientation), relationship and family-care status, academic information (institution type, academic role, and rank), remote work status, and professional or career-related setbacks. Following the consent agreement, two gating questions were used to establish inclusion criteria which required participants to work at a 2- or 4-year college, university, or academic research institution within the United States within the discipline of biomedical or life sciences.

Participant Recruitment

We utilized purposive and snowball sampling strategies to obtain a representative sample of biomedical scientists within the United States. First, we utilized publicly available data provided by the National Institutes of Health to obtain email addresses of 56,607 principal investigators supported by NIH extramural grants in fiscal year 2018[1]. The list of principal investigators was randomized and 21,500 individuals received an email invitation to participate in the survey. In order to specifically recruit postdoctoral fellows, an invitation to participate in the survey was distributed via email to 10,076 members of the National Postdoctoral Association. In addition, 43 HHMI Hanna H. Gray Fellows and 28 postdoctoral fellows supported by Institutional Research and Academic Career Development Awards were invited to participate in the survey via email addresses obtained from publicly-accessible program webpages and institutional directories.

All groups received the same invitation to participate in the survey which encouraged them to share the survey with others within their professional network. Invitations were distributed and responses were collected using Qualtrics™ software between October 1st, and November 30th, 2020. Of the 31,647 email invitations sent, 30,330 (96%) were successfully delivered. However, emails distributed from Qualtrics™ can be flagged as spam or junk and might never be viewed by the intended recipients. A total of 635 individuals met the inclusion criteria and completed the survey for an approximate response rate of 2%.

Resilience Measurement

We utilized the 10-Item abbreviation of the Connor-Davidson Resilience Scale (CD-RISC-10) to assess biomedical scientists' resilience. The CD-RISC-10 measures *overall* resilience, conceptualized as one's capacity to adapt to and bounce back from adversity.[2] Respondents rate their attainment of each of ten indicators of resilience (e.g., "able to adapt when changes occur") on a 5-point Likert scale ranging from 0 ("not true at all") to 4 ("true nearly all the time"). The total CD-RISC-10 score (minimum=0, maximum=40) is obtained by summing up scores on the constituent items. Higher CD-RISC-10 scores indicate greater resilience.

© 2023 Woitowich NC et al. *JAMA Network Open.*

Respondent Career Stage

Respondents indicated if they were postdoctoral fellows or faculty. Faculty respondents shared their tenure eligibility (non-tenure eligible or tenure-eligible) and faculty rank (assistant, associate, or full professor). We coded respondents as being early career if they were postdoctoral fellows, assistant professors, or non-tenure eligible faculty. We chose to include non-tenure eligible faculty in this category due to the unique pressures they may face in career and economic uncertainty.[3] Respondents were coded as mid-to-late career if they were tenure-eligible, associate or full professors.

Data Analyses

Data were analyzed using Graphpad Prism® software version 9. Descriptive statistics were calculated for all variables of interest. Categorical variables were summarized with frequencies and percentages, and continuous variables were summarized with medians and interquartile ranges.

Regression analysis

In the professional setback model, the dependent variable is a dichotomous indicator of whether a respondent experienced or did not experience a professional setback during the Covid-19 pandemic. Logistic regression models were fitted on respondent gender (binary categories of male vs non-male, with male as the reference), CD-RISC-10 score, and the binary yes/no variable of engaging in additional child/elder care work responsibilities during the pandemic (with no additional care work as the reference). Odds ratios and their 95% confidence intervals are used to summarize the logistic regression model findings.

Supplemental References

1. National Institutes of Health. Annual listing of Mailing and Email Addresses of Principal Investigators Supported by NIH Extramural Grants for FY 2018. Accessed October 1, 2020. https://www.nih.gov/sites/default/files/institutes/foia/FY-18-mailing-email-eddresses-principal-investigators-61-19-CS-LLs-01242019-approved.xlsx

2. Campbell-Sills, L., & Stein, M. B.; Psychometric Analysis and Refinement of the Connor–Davidson Resilience Scale (CD-RISC): Validation of a 10-item Measure of Resilience; *Journal of Traumatic Stress* 2007; 20(6):1019–1028. https://doi.org/10.1002/jts.20271

3. Culver K, Kezar A. *The Impacts of 2020 on Advancement of Non-Tenure- Track and Adjunct Faculty*. Accessed June 16, 2023. https://nap.nationalacademies.org/resource/26405/6_The_Impacts_of_2020_on_Advancement_of_Contingent_Faculty-Culver_Kezar.pdf

© 2023 Woitowich NC et al. *JAMA Network Open.*
