# Supplementary material for: Evaluation of Professional Setbacks and Resilience in Biomedical Scientists During the COVID-19 Pandemic
Source: JAMA Netw Open. 2023 Aug 9;6(8):e2328027. doi: 10.1001/jamanetworkopen.2023.28027 (PMC10413169; doi:10.1001/jamanetworkopen.2023.28027)
Supplement: Supplement 2. — Data Sharing Statement [file jamanetwopen-e2328027-s002.pdf]

Data Sharing Statement

Woitowich. Evaluation of Professional Setbacks and Resilience in Biomedical Scientists During the COVID-19 Pandemic. *JAMA Netw Open*. Published August 09, 2023. doi:10.1001/jamanetworkopen.2023.28027

Data
Data available: Yes
Data types: Deidentified participant data
How to access data: Data can be obtained by emailing the corresponding author (nicole.woitowich@northwestern.edu)
When available: With publication

Supporting Documents
Document types: None

Additional Information
Who can access the data: Anyone requesting the data
Types of analyses: Any purpose
Mechanisms of data availability: Data will be made freely available and without undue reservation.
